# Recent Progress in the Synthesis, Design, and Electrochemical Applications of Porphyrin/Phthalocyanine-Based Metal–Covalent Organic Frameworks

**DOI:** 10.3390/nano16161036

**Published:** 2026-08-20

**Authors:** Peng Huang, Gaowei Xue, Chengfeng Jiang, Li Hu, Jiahui Yuan, Qiang Huang, Hongxing Jia

**Affiliations:** 1 College of Materials Science and Engineering, National Engineering Research Center for Magnesium Alloys, Chongqing University, Chongqing 400044, China; huangpeng@stu.cqu.edu.cn (P.H.); xuegaowei@stu.cqu.edu.cn (G.X.); jiangcf@stu.cqu.edu.cn (C.J.); 2 Chongqing Institute of New Energy Storage Materials and Equipment, Chongqing 401135, China; 3 College of Physics, Center of Quantum Materials and Devices, Chongqing University, Chongqing 401331, China; cqu_hl@163.com; 4 The McComish Department of Electrical Engineering and Computer Science, Jerome J. Lohr College of Engineering, South Dakota State University, Brookings, SD 57007, USA; jiahui.yuan@jacks.sdstate.edu; 5 School of Chemistry and Chemical Engineering, Nantong University, Nantong 226019, China

**Keywords:** metal–covalent organic frameworks, porphyrin, phthalocyanine, synthesis strategies, electrochemical energy storage

## Abstract

The limitations of conventional inorganic electrodes call for organic alternatives for advanced energy storage. Metal–covalent organic frameworks (MCOFs) integrate the metal active sites of metal–organic frameworks (MOFs) with the high chemical stability imparted by strong covalent bonds in covalent organic frameworks (COFs) while retaining the high specific surface area and tunable porosity of both material classes. Among these, MCOFs constructed from porphyrin and phthalocyanine building units have emerged as a research hotspot in electrochemical energy storage owing to their inherent 18π-conjugated macrocyclic electronic systems, well-defined M–N_4_ coordination sites, and potential bipolar charge storage characteristics. This review systematically summarizes recent advances in this class of materials. First, from the perspective of metal center introduction timing, three core synthetic strategies—pre-metallation, simultaneous metallation, and post-metallation—are categorized and evaluated in terms of coordination precision, synthetic efficiency, and scalability potential. Second, the regulatory effects of two-dimensional layered and three-dimensional interpenetrated structures on charge transport pathways and structural stability are elucidated. Subsequently, the applications of porphyrin/phthalocyanine-based MCOFs in lithium-based batteries, zinc-based batteries, sodium/potassium-ion batteries, and supercapacitors are reviewed in detail, with emphasis on the key roles of metal active sites in catalytic conversion, chemical anchoring/confinement, interface stabilization, and pseudocapacitive contribution. Finally, future directions to address key performance and mechanistic bottlenecks are discussed. This review aims to provide a systematic reference for the rational design and energy storage applications of high-performance porphyrin/phthalocyanine-based MCOFs.

## 1. Introduction

The rapid development of portable electronic devices, electric vehicles, and smart grid technologies has created an urgent demand for high-performance, low-cost, and environmentally friendly electrochemical energy storage systems. Among the current mainstream energy storage technologies, lithium-ion batteries (LIBs) have achieved dominance due to their high energy density, long cycle life, and fast charge–discharge capabilities [1,2]. However, the limited and unevenly distributed global lithium reserves have driven up costs, while the performance of conventional inorganic electrode materials—such as transition metal oxides and graphite—is approaching theoretical limits, making it difficult to meet the requirements of future high-energy and high-safety storage systems [3,4]. Consequently, there is an urgent need to develop novel electrode material systems with abundant resources, tunable structures, and designable performance characteristics. Organic electrode materials have attracted extensive attention in recent years owing to their high elemental abundance, structural tunability, environmental friendliness, and recyclability [5,6]. Among the various organic framework materials, covalent organic frameworks (COFs) and metal–organic frameworks (MOFs) represent two prominent classes of porous crystalline materials. COFs are constructed via strong covalent bonds between organic building units, endowing them with excellent chemical and thermal stability, high specific surface area, and regularly tunable pore structures [7,8]. However, their purely organic skeletons lack metal active sites, limiting their application in scenarios requiring metal-centered electrocatalytic transformations and multi-electron reactions [9]. MOFs, in contrast, are self-assembled through coordination bonds between metal ions/clusters and organic ligands, enabling the introduction of abundant metal active sites and diverse topological structures [10,11]; nevertheless, their coordination bonds are susceptible to cleavage under harsh conditions such as strong acids, strong bases, or high temperatures, resulting in insufficient stability [12,13]. Apart from pristine MOFs and COF systems, pyrolyzed derivatives of MOFs/COFs have also been widely explored for energy storage, yet high-temperature carbonization inevitably disrupts coordination structures and causes metal aggregation [14,15].

The emergence of metal–covalent organic frameworks (MCOFs) effectively integrates the advantages of both material classes. By introducing metal ions into the COF framework through coordination bonds, MCOFs retain the high specific surface area, excellent stability, and designable porosity of COFs while incorporating the metal active sites of MOFs [16,17]. The synergistic interplay between metal centers and covalent backbones endows MCOFs with unique electronic structures and catalytic functions, rendering them promising for applications in electrocatalysis [18,19], energy storage [20], photocatalysis [21,22], and sensing [23]. Among the various available building blocks, porphyrins and phthalocyanines stand out as ideal platforms for constructing high-performance MCOFs electrode materials owing to their unique molecular and electronic structures. These macrocyclic molecules offer three core advantages: (i) their intrinsic 18π-conjugated electronic systems provide excellent charge delocalization and electron transport capabilities [24]; (ii) the square-planar arrangement of four pyrrole/isoindole nitrogen atoms within the macrocyclic cavity allows for the precise coordination of diverse metal ions to form stable M–N_4_ sites, with the coordination environment fixed by the covalent macrocyclic framework, offering both structural certainty and electronic tunability [25]; and (iii) the electron-rich π systems confer both p-type (anion storage) and n-type (cation storage) doping potential, providing a molecular basis for bipolar charge storage [26]. Furthermore, the diverse peripheral substituent modifications available for porphyrins/phthalocyanines enable the fine-tuning of band structures, solubility, and assembly behavior, offering ample space for function-oriented molecular design. These combined attributes, which are not simultaneously available in other macrocyclic systems, and the extensive electrochemical energy storage data accumulated for this family, collectively justify their selection as the exclusive focus of this review. The rigid square-planar M–N_4_ geometry of porphyrins/phthalocyanines also distinguishes them from other metal coordination environments commonly found in conventional MOFs. In contrast to asymmetric M–N_x_ or ionic M–O_x_ sites, the symmetric and covalently confined M–N_4_ cavity ensures structural integrity during repeated redox cycling and delivers favorable d-band centers for catalytic intermediate adsorption; these features, combined with the fully delocalized 18π system discussed above, underpin both the electrochemical stability and the catalytic/capacitive functionality of this material family.

In recent years, porphyrin/phthalocyanine-based MCOFs have made significant progress in lithium-ion batteries, lithium-sulfur batteries, lithium-carbon dioxide batteries, zinc-air batteries, zinc-iodine batteries, sodium/potassium-ion batteries, and supercapacitors. Nevertheless, no dedicated review has systematically elucidated the structure–property relationship chain for porphyrin/phthalocyanine-based MCOFs in the context of electrochemical energy storage. Several recent reviews have covered metal-containing COFs and macrocycle-based frameworks from the perspectives of general materials chemistry [27], catalysis [28], sensor applications [29], or broad COFs/MCOFs design principles [30]; others have focused on general MCOFs structural design [31] or small-molecule/polymer organic electrodes [32,33]. However, none has provided an in-depth, systematic analysis specifically dedicated to porphyrin/phthalocyanine-based MCOFs for electrochemical energy storage, particularly regarding their metallation strategies, dimensional regulation, and battery-specific performance bottlenecks. This gap underscores the need for the present review. More critically, most current studies remain confined to the “material synthesis + performance testing” paradigm, with insufficient understanding of core scientific questions such as the dynamic evolution mechanisms of metal active sites during charge–discharge processes, the synergistic relationship between framework structure and ion transport, and the intrinsic causes of material degradation.

Against this backdrop, this review focuses on the research progress of porphyrin/phthalocyanine-based MCOFs in electrochemical energy storage. First, three core synthetic strategies are systematically summarized from the perspective of metal center introduction timing, with an analysis of their respective advantages and limitations in terms of coordination precision, synthetic efficiency, and scalability potential. Second, the regulatory effects of two-dimensional layered and three-dimensional interpenetrated structures on charge transport and stability are elucidated. Subsequently, the application progress of these materials in various energy storage systems is reviewed in detail, with emphasis on the multiple functional mechanisms of metal active sites. Finally, several future breakthrough directions are proposed to address the current performance bottlenecks and knowledge gaps. This review aims to provide a systematic reference for the rational design and energy storage applications of high-performance porphyrin/phthalocyanine-based MCOFs.

## 2. Synthesis Strategies for Porphyrin/Phthalocyanine-Based MCOFs

The synthesis of porphyrin/phthalocyanine-based MCOFs revolves around addressing two key challenges: first, the efficient formation of COFs (via imine condensation, imidization, dioxin bond formation, etc.), and second, the successful incorporation of metal ions into the macrocyclic cavity either during or after framework formation to establish stable M–N_4_ coordination configurations. Based on the timing of metal center introduction, existing synthetic strategies can be categorized into three types: pre-metallation, simultaneous metallation, and post-metallation. These three strategies each offer distinct advantages and limitations in terms of coordination precision, synthetic efficiency, and scalability potential, making them suitable for different research objectives and material systems.

### 2.1. Pre-Metallation

The pre-metallation strategy involves coordinating metal ions with porphyrin/phthalocyanine ligands to form “metal-macrocycle monomers” prior to COF framework construction; these monomers are then used as functional building units in subsequent covalent condensation reactions. In this process, the metal center becomes an intrinsic component of the monomer, with its coordination environment fixed at the monomer synthesis stage. The most prominent advantage of this strategy is that metal coordination can be completed independently before framework construction, effectively avoiding the influence of subsequent condensation reaction conditions on the coordination process. Through conventional metal complex synthesis methods (e.g., refluxing metal salts with macrocyclic ligands), the metallation conditions can be fully optimized, facilitating high metal loading and uniform coordination geometries.

**Figure 1:**
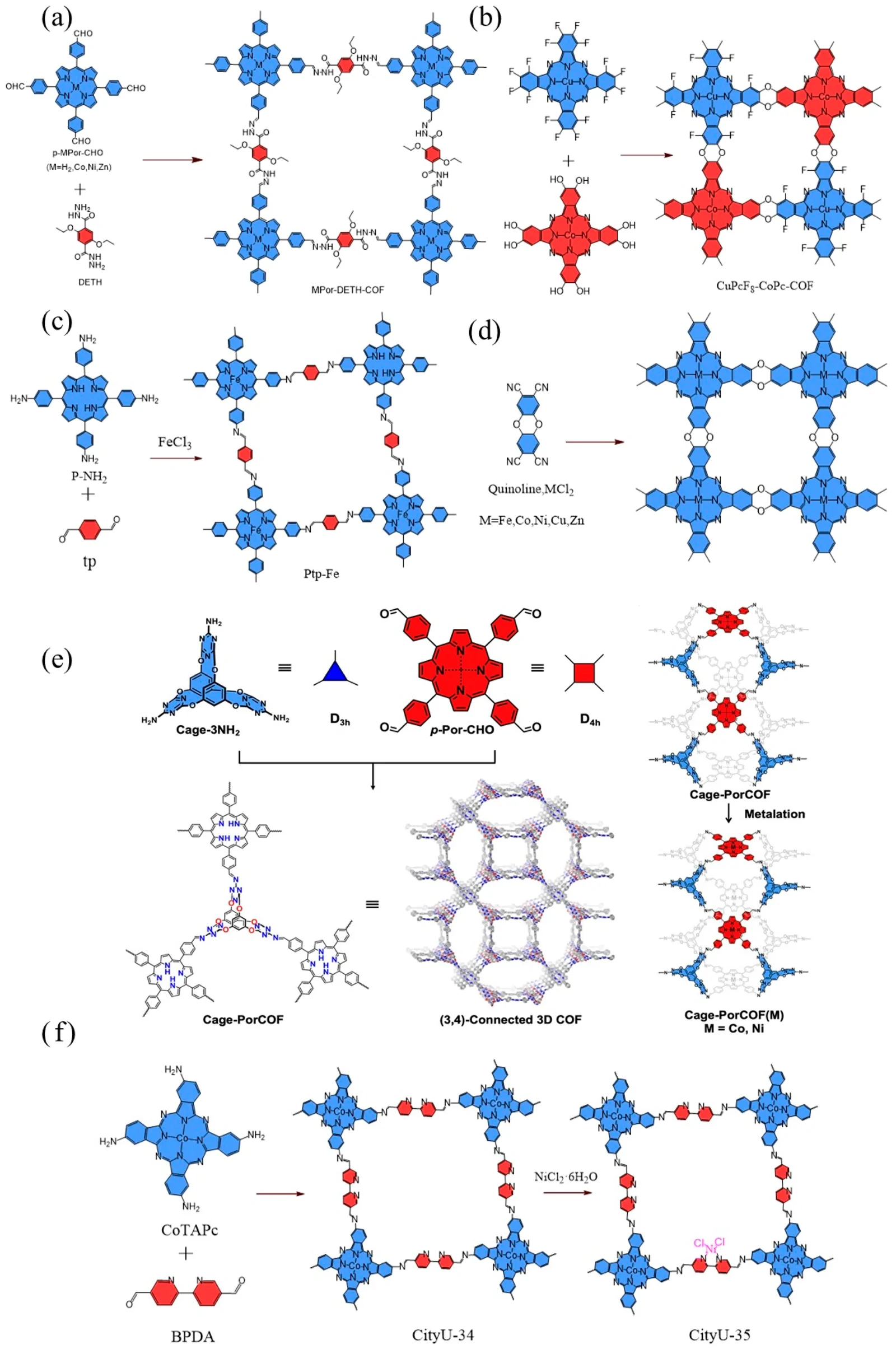
(**a**) Schematic synthesis of MPor-DETH-COF. (**b**) Typical solvothermal synthesis of CuPcF_8_-CoPc-COF. (**c**) Schematic synthesis of Ptp-Fe. (**d**) Construction diagram of MPc-based COFs. (**e**) Schematic synthesis of Cage-Por COF, Cage-Por COF(Co), and Cage-Por COF(Ni) [42]. Copyright 2025 Wiley-VCH GmbH. (**f**) Schematic synthesis of CityU-34 and CityU-35.

In the pre-metallation synthesis of porphyrin-based MCOFs, Wang et al. [34] employed a homogeneous direct synthesis approach, first preparing porphyrin aldehyde monomers containing metal ions such as Co^2+^, Ni^2+^, and Zn^2+^, then subjecting them to solvothermal condensation with 2,5-diethoxyterephthalohydrazide (DETH) in a 1,2-dichlorobenzene/n-butanol/acetic acid mixed solvent at 120 °C for 3–7 days, yielding hydrazone-linked two-dimensional porphyrin-based MCOFs (MPor-DETH-COF, M = Co, Ni, Zn) (Figure 1a). This series of materials maintained highly consistent two-dimensional layered structures and well-ordered pores, with uniformly distributed metal centers throughout the framework. Fang et al. [35] adopted a liquid–air interfacial polymerization strategy, using meta-benzohydrazide-substituted metalloporphyrins (M = Co, Cu, Zn, Mg, etc.) as core monomers and trialdehyde linkers (TOB, TFPA, TFB) in dimethyl sulfoxide (DMSO). The amphiphilic nature of the metalloporphyrins drove their spontaneous aggregation at the liquid–air interface formed between DMSO and moist air, where condensation reactions generated dynamic covalent acylhydrazone bonds, ultimately yielding defect-free self-standing COF films (CoP-TOB, CoP-TFPA, CoP-TFB). This method circumvents the high-temperature, prolonged reaction times typical of traditional solvothermal conditions, offering a mild and efficient alternative pathway for pre-metallation.

The pre-metallation strategy is equally applicable to phthalocyanine-based systems and their coupled porphyrin hybrids. Huang et al. [36] pre-synthesized Cu^2+^-containing hexadecafluorophthalocyanine copper (CuPcF_16_) and Co^2+^-containing octahydroxyphthalocyanine cobalt (CoPc (OH)_8_), then employed nucleophilic aromatic substitution reactions in DMF with triethylamine as the catalyst at 100 °C to construct dioxin-linked covalent frameworks, achieving the precise introduction of both Cu and Co dual-metal sites in one step. The resulting bimetallic polyphthalocyanine COFs (CuPcF_8_-CoPc-COF and CuPcF_8_-CoNPc-COF) (Figure 1b) exhibited excellent structural robustness under harsh conditions. Peng et al. [37] used pre-coordinated CoTAPc (containing Co-N_4_ sites) as the metallo-organic node, reacting it with two different porphyrin linkers—5,15-bis(4-aminophenyl)-10,20-diphenylporphyrin (DAPor) and 5,10,15,20-tetrakis(4-aminophenyl)porphyrin (TAPor)—in N-methylpyrrolidone/n-butanol with isoquinoline as the catalyst at 180 °C via imidization, yielding two phthalocyanine-porphyrin coupled COFs (CoPc-2H_2_Por and CoPc-H_2_Por) with AA stacking and different pore sizes and conjugation degrees. This design synergistically combines the high conjugation of phthalocyanines with the structural tunability of porphyrins, offering new avenues for constructing multifunctional MCOFs.

Notably, pre-metallation requires that the metal-macrocycle monomers exhibit good solubility in the COF condensation solvent system and maintain structural stability under high-temperature condensation conditions. Additionally, the extra steps for metal complex synthesis and purification render the overall procedure relatively cumbersome, with prolonged synthesis times and higher costs. This strategy is best suited for mechanistic studies requiring stringent homogeneity of active sites, though its scalability potential remains limited.

### 2.2. Simultaneous Metallation

The simultaneous metallation strategy involves placing metal salts, porphyrin/phthalocyanine precursors, and bridging linkers together in the same reaction system, where COF framework construction and metal ion coordination within the macrocyclic cavity occur concurrently. Metal salts participate directly in the reaction without the need for pre-synthesized metal-macrocycle monomers. The most prominent advantage of this strategy lies in its procedural simplicity and operational convenience—metal coordination and framework crystallization are accomplished in a single process, significantly shortening synthesis cycles and reducing raw material costs while improving preparation efficiency.

In the simultaneous metallation synthesis of porphyrin-based MCOFs, Kaczmarek et al. [38] developed an “assembler” strategy by introducing pyrazine as a template pre-orientation unit into the COF-366 synthesis system. Pyrazine cooperates with octahedrally coordinated metal ions (e.g., Fe^2+^, Cu^2+^) to guide porphyrin linkers away from random aggregation caused by self-polymerization, markedly improving the crystallinity and porosity of the one-pot product. The resulting Co@COF-366 achieved a BET surface area of approximately 1200 m^2^ g^−1^. Lin et al. [39] used 5,10,15,20-tetrakis(4-aminophenyl)porphyrin (P-NH_2_) and terephthalaldehyde (tp) as monomers, simultaneously introducing FeCl_3_ into the imine condensation system. Fe^3+^ served a dual role: coordinating with porphyrin rings to form Fe–N_4_ sites, while the HCl generated by Fe^3+^ hydrolysis protonated uncoordinated porphyrin units, achieving simultaneous metallation and protonation dual functionalization (Figure 1c).

For phthalocyanine-based systems, Chen et al. [40] developed a versatile one-pot condensation strategy in which tetracyano precursors (DDTC, BBTC) were converted by MCl_2_ (M = Ni, Zn, Fe, Co, Cu) in quinoline at 230 °C for 18 h into phthalocyanine macrocycles with simultaneous M–N_4_ coordination and dioxin-linked two-dimensional framework formation. This method produced AA stacking metallophthalocyanine COFs (MPc-nDXI-COFs) in 80–96% yields (Figure 1d).

The simultaneous metallation strategy offers significant advantages in synthetic efficiency, though several limitations remain. Competitive coordination among different metal ions may lead to uneven distribution, and the presence of metal ions can sometimes affect the COF crystallization kinetics, requiring fine optimization of solvent, temperature, and other conditions. Moreover, the solubility of metal salts in the reaction medium imposes additional constraints. This strategy is particularly suitable for rapid material screening and scalable preparation scenarios where synthetic efficiency is paramount.

### 2.3. Post-Metallation

The post-metallation strategy involves first synthesizing a metal-free or partially metalated porphyrin/phthalocyanine COF precursor, then introducing target metal ions into the macrocyclic cavity through a separate metallation reaction to form the final MCOF. The core advantage of this strategy lies in its operational flexibility and functional diversity: researchers can first prepare high-quality metal-free COF precursors under optimized condensation conditions, then react them with different metal salts separately to rapidly construct a series of isostructural MCOFs for performance comparison. Additionally, this strategy is suitable for cases where metal-macrocycle monomers exhibit poor solubility or instability under COF condensation conditions, as well as for constructing complex systems requiring the introduction of multiple metals.

In the post-metallation synthesis of porphyrin-based MCOFs, Liu et al. [41] first synthesized a porphyrin COF (H_2_-TPCOF) ultrathin nanosheet via solvothermal imine condensation using metal-free 5,10,15,20-tetrakis(4-aminophenyl)porphyrin (TAPP) and non-linear 2,6-pyridinedicarbohydrazide (PCBA), then refluxed it with cobalt acetate or copper acetate to coordinate metal ions with porphyrin N atoms, yielding Co-TPCOF and Cu-TPCOF. This study achieved the introduction of different metals using the same precursor. Li et al. [42] extended the strategy to three-dimensional systems: a non-interpenetrated porous 3D COF (Cage-PorCOF) was first constructed from a threefold-symmetric nitrogen-rich organic cage and a fourfold-symmetric porphyrin aldehyde, then reacted with cobalt acetate or nickel acetate in ethanol to coordinate Co^2+^ or Ni^2+^ with porphyrin N atoms, forming M–N_4_ sites in Cage-PorCOF(Co) and Cage-PorCOF(Ni) (Figure 1e). This work validated the feasibility of the post-metallation strategy in 3D COF systems.

Post-metallation can also be employed to construct bimetallic MCOFs containing two different coordination sites. Zhang et al. [43] first condensed cobalt phthalocyanine tetraamine with 2,2′-bipyridine-5,5′-dicarboxaldehyde (BPDA) to prepare a 2D COF (CityU-34) containing Co–N_4_ sites while reserving bipyridine coordination sites in the framework. Subsequent reaction with NiCl_2_ allowed Ni^2+^ to coordinate with bipyridine N atoms, introducing a second metal without disrupting the existing Co–N_4_ sites, yielding the bimetallic COF CityU-35 (Figure 1f). This “pre-embedded coordination site” strategy achieves site-specific introduction of target metals, avoiding random distribution.

The post-metallation strategy also suffers from several limitations. First, metal ions must diffuse from the precursor’s pores or interlayers to the macrocyclic cavity coordination sites; high crystallinity or narrow pores may result in incomplete coordination or low metal loading. Second, post-introduced metals may preferentially adsorb on the COF surface or at defect sites, compromising active site uniformity. Furthermore, metallation reaction conditions (temperature, solvent, reaction time) must be carefully controlled to avoid damaging the existing COF framework. For quick reference, the key features of the three metallation routes are summarized in Table 1. Notably, although the three metallation routes have been individually validated, systematic quantitative comparisons of their crystallinity and defect density are currently lacking in the literature. Most studies prioritize demonstrating the feasibility of individual strategies over benchmarking structural quality across different synthetic approaches, and this gap represents an important direction for future investigation.

## 3. Dimensional Regulation

The dimensional structure of MCOFs significantly affects their charge transport pathways, active site accessibility, and structural stability. While synthetic strategies address “how to introduce metals into the framework”, dimensional regulation determines the spatial arrangement of metal active sites and their interaction modes with guest molecules. Two-dimensional layered structures facilitate in-plane conjugated electron transport, with interlayer π–π stacking providing additional conductive channels; however, dense stacking may also impede ion intercalation. Three-dimensional interpenetrated structures enhance framework stability through spatial interlocking, and their interpenetrated networks can provide multidirectional ion transport pathways, though the multi-directional charge migration paths often result in reduced apparent electronic conductivity. Therefore, rational selection of dimensional structures based on target application scenarios is a critical factor in harnessing the energy storage potential of porphyrin/phthalocyanine-based MCOFs.

### 3.1. 2D Structures

**Figure 2:**
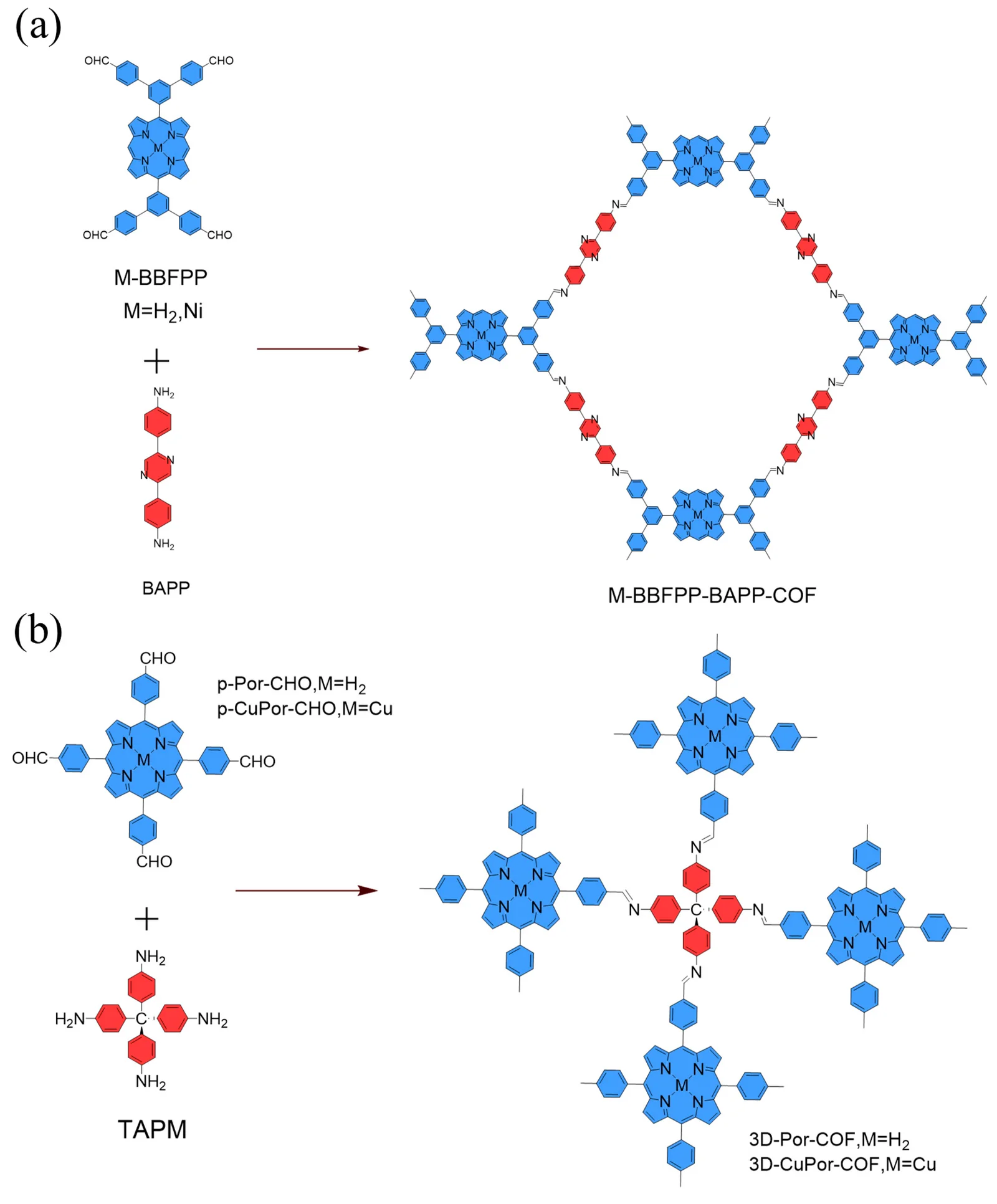
(**a**) Synthesis of M-BBFPP-BAPP-COF (M=H_2_, Ni). (**b**) Schematic synthesis of 3D-Por-COF and 3D-CuPor-COF.

Two-dimensional (2D) porphyrin/phthalocyanine-based MCOFs represent the most extensively studied structural type. Their layered morphology endows materials with large specific surface areas and exposed active sites, facilitating charge transport and ion diffusion. Huang et al. [44] employed symmetry combination strategies of “D_2_h + C_2_” and “D_2_h + D_2_h” using bis [3,5-bis(4-formylphenyl)phenyl]porphyrin (BBFPP, metal-free or Ni-pre-metallated) as the porphyrin node, paired with 2,5-bis(4-aminophenyl)pyrazine (BAPP, C_2_-symmetric) and 2,3,5,6-tetrakis(4-aminophenyl)pyrazine (TAPP, D_2_h-symmetric) as linkers, respectively, to construct 2D COFs with sql and bex topologies via solvothermal condensation (BBFPP-BAPP-COF and BBFPP-TAPP-COF) (Figure 2a). Comparative studies revealed that the denser in-plane connections in the bex topology enhanced framework conjugation, improving charge transport efficiency and providing model systems for understanding the influence of 2D COF topology on electronic behavior. Jiang et al. [45] used C_4_-symmetric nickel octacarboxyphthalocyanine tetraanhydride (NiTAPc) and C_2_-symmetric bipyridine diamine (Bpyd) as building units to prepare AA stacking 2D phthalocyanine COFs (2D-NiPc-COF) via imidization. In this structure, Ni–N_4_ sites are periodically arranged within layers, with adjacent layers providing additional charge transport channels through π–π stacking of phthalocyanine macrocycles. This “in-plane conjugation + interlayer stacking” synergistic model provides a structural foundation for optimizing the conductivity of 2D MCOFs.

### 3.2. 3D Structures

Three-dimensional (3D) MCOFs have been less extensively studied, yet their unique spatial structures offer potential advantages in stability and pore openness. Wang et al. [46] synthesized the first 3D porphyrin COF (3D-CuPor-COF) via imine condensation using tetrahedral tetrakis(4-aminophenyl)methane (TAPM) and 5,10,15,20-tetrakis(4-formylphenyl)copper porphyrin (p-CuPor-CHO) as monomers (Figure 2b). This material exhibited high specific surface area and good thermal stability, validating the feasibility of using porphyrin units to construct 3D COFs. Han et al. [47] further used phthalocyanine tetraanhydrides (MTAPc, M = Co^2+^ or H_2_) and 1,3,5,7-tetrakis(4-aminophenyl)adamantane (TAPA) as building units to construct pts-topology 3D COFs (MPc-PI-COF-3) via imidization. The structure consists of interpenetrated pts networks with interconnected pores; the interpenetration enhances framework mechanical stability through steric hindrance. Although 3D structures are generally inferior to 2D systems in electron transport, their more open pore environments favor the diffusion of large guest molecules, offering unique value in high-loading energy storage systems.

In summary, 2D and 3D structures each have their own emphases: 2D systems leverage the charge transport advantages provided by in-plane conjugation and interlayer stacking, making them more suitable for energy storage scenarios requiring high-rate performance; 3D systems utilize the framework stability conferred by spatial interlocking, along with multidirectional ion transport pathways achievable under appropriate structural design, holding application potential in energy storage systems requiring long cycle life and high mass loading. Future work may focus on predicting dimensional structures at the molecular design stage through the rational selection of monomer geometries and connection modes, combined with the systematic optimization of synthetic conditions for controllable construction.

## 4. Applications in Electrochemical Energy Storage

The application of porphyrin/phthalocyanine-based MCOFs in electrochemical energy storage benefits from the synergy between their unique molecular structures and framework properties. On the one hand, the macrocyclic M–N_4_ sites provide high-density metal active centers that can directly contribute to charge storage capacity through metal ion valence changes or serve as catalytic centers for accelerating multi-electron conversion reactions (such as polysulfide conversion, oxygen reduction/evolution, and CO_2_ reduction). On the other hand, the ordered pores and tunable structures of the COF framework provide a structural basis for ion transport and active species confinement. Furthermore, two-dimensional layered or three-dimensional interpenetrated structures can further modulate charge transport pathways and structural stability. Leveraging these advantages, this class of materials has demonstrated broad application prospects in lithium-based batteries, zinc-based batteries, sodium/potassium-ion batteries, and supercapacitors.

### 4.1. Lithium-Based Batteries

#### 4.1.1. Lithium-Ion Batteries

Lithium-ion batteries represent the most mature electrochemical energy storage technology; however, the capacity of conventional graphite anodes is approaching its theoretical limit (372 mAh g^−1^) [48,49], necessitating the development of high-capacity, long-cycle-life anode materials. Organic framework materials have attracted attention due to their structural designability and resource sustainability, but the poor conductivity and low active site utilization of COFs have limited their application, in contrast to inorganic anode systems that typically rely on element doping to enhance conductivity and stability [50]. Porphyrin/phthalocyanine-based MCOFs offer a viable pathway to overcome these bottlenecks by introducing macrocyclic conjugated systems to enhance charge transport, while utilizing M–N_4_ metal active centers to participate in redox reactions and contribute additional capacity.

**Figure 3:**
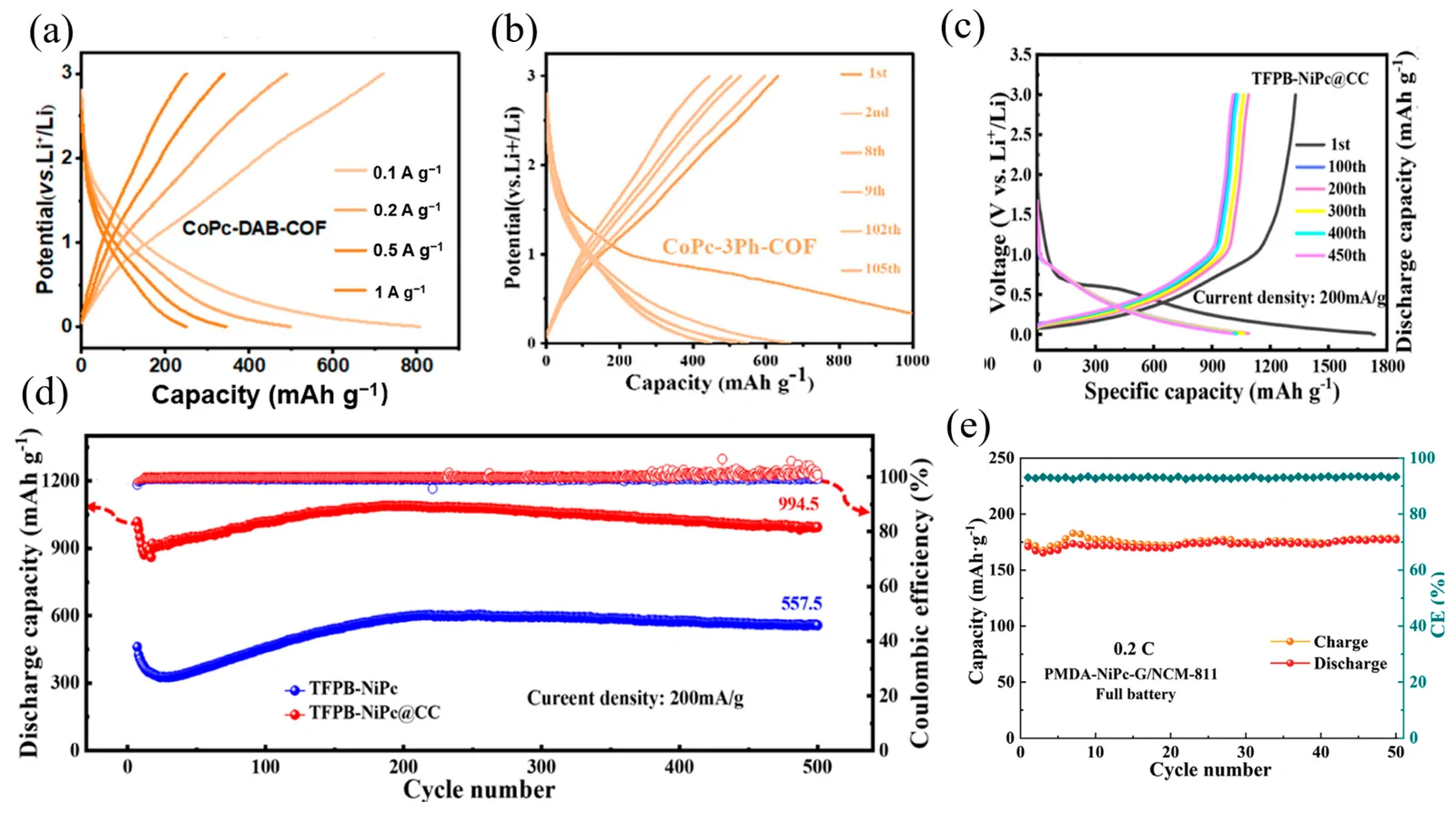
(**a**) Charge–discharge curves of CoPc-DAB-COF electrodes [51]. Copyright 2025 American Chemical Society. (**b**) The charge–discharge curves of the CoPc-3Ph-COF at 100 mA g^−1^ [52]. Copyright 2024 Wiley-VCH GmbH. (**c**) Charge–discharge curves of TFPB-NiPc@CC. (**d**) Long-cycle plots of TFPB-NiPc and TFPB-NiPc@CC electrodes at 200 mA g^−1^ current density [53]. Copyright 2025 American Chemical Society. (**e**) Cycle performance of PMDA-NiPc-G/NCM-811 full battery (0.2 C) [54]. Copyright 2023 Wiley-VCH GmbH.

In phthalocyanine-based MCOFs, Jiang et al. [51] synthesized two piperazine-linked conjugated phthalocyanine MCOFs (CoPc-BTM-COF and CoPc-DAB-COF), both featuring sql topology and AA stacking, with pore sizes of 1.62 nm and 1.90 nm, respectively. At 100 mA g^−1^, these materials delivered reversible capacities of 877 and 669 mAh g^−1^ (Figure 3a). The same group further synthesized CoPc-Ph-COF and CoPc-3Ph-COF with pore sizes of 1.6–2.4 nm, among which CoPc-3Ph-COF achieved a reversible capacity of 1086 mAh g^−1^ at 100 mA g^−1^ (Figure 3b), attributed to the superior conductivity imparted by its fully conjugated framework [52].

Combining phthalocyanine MCOFs with highly conductive carbon substrates is an effective strategy for enhancing electrochemical performance. Chen et al. [53] grew phthalocyanine COFs (TFPB-NiPc) on carbon cloth via in situ Schiff-base reactions, obtaining a binder- and conductive-additive-free self-supporting composite anode TFPB-NiPc@CC. This electrode delivered a specific capacity of 1090.2 mAh g^−1^ at 200 mA g^−1^ (Figure 3c), maintaining 994.5 mAh g^−1^ after 500 cycles (Figure 3d). Li et al. [54] composited a multi-carbonyl-containing phthalocyanine MCOF (PMDA-NiPc) with graphene via the solvothermal reaction. Graphene served dual roles as a conductive medium to enhance electron transport and as a dispersing agent to reduce COF agglomeration, forming small-volume, few-layer structures that shortened ion migration pathways. The resulting PMDA-NiPc-G composite anode achieved a reversible capacity of 1861 mAh g^−1^ at 100 mA g^−1^ after 600 cycles (based on PMDA-NiPc mass), with a Li^+^ diffusion coefficient 3.6 times that of the pristine COF. Full cells assembled with NCM-811 or LFP cathodes exhibited capacity retentions of 60.2% and 74.7% after 200 cycles at 1 C, respectively; the PMDA-NiPc-G/NCM-811 full cell maintained approximately 100% capacity retention after 50 cycles at 0.2 C (Figure 3e). Pan et al. [55] coated PMDA-NiPc onto nano-silicon particles, utilizing the strong confining and buffering effects of the COF layer to effectively suppress silicon volume expansion. This composite anode delivered a reversible capacity of 654 mAh g^−1^ after 2000 cycles at 5 A g^−1^, with the full cell retaining 85.8% capacity after 100 cycles at 0.2 C.

Collectively, the application potential of phthalocyanine-based MCOFs in lithium-ion battery anodes can be summarized in three aspects: (i) the intrinsic electron transport channels provided by the macrocyclic conjugated framework—fully conjugated structures effectively reduce charge transport resistance; (ii) the redox reactions involving M–N_4_ sites—metal center valence changes directly contribute additional capacity; and (iii) the facilitation of Li^+^ transport by ordered pores—well-defined pore structures promote rapid ion diffusion. Current studies have preliminarily validated the feasibility of this material class, though further research is needed on improving intrinsic conductivity, constructing high-loading electrodes, and elucidating metal site evolution mechanisms during charge–discharge cycling. In comparison, porphyrin-based MCOFs have been less frequently reported in this scenario, though their potential advantages may lie in the richer redox sites of the porphyrin ring and the more flexible molecular design space.

#### 4.1.2. Lithium Metal Batteries

Lithium metal anodes are considered ideal negative electrodes for next-generation high-energy-density batteries due to their extremely high theoretical specific capacity (3860 mAh g^−1^) and lowest electrochemical potential [56,57]. However, the uncontrolled growth of lithium dendrites not only poses safety risks by potentially piercing the separator, but also exacerbates side reactions with the electrolyte due to their high surface area, leading to the continuous consumption of active lithium and electrolyte, which in turn results in low Coulombic efficiency and shortened cycle life [58]. Constructing artificial protective layers on lithium anodes to regulate uniform Li^+^ deposition is an effective strategy for suppressing dendrite formation.

**Figure 4:**
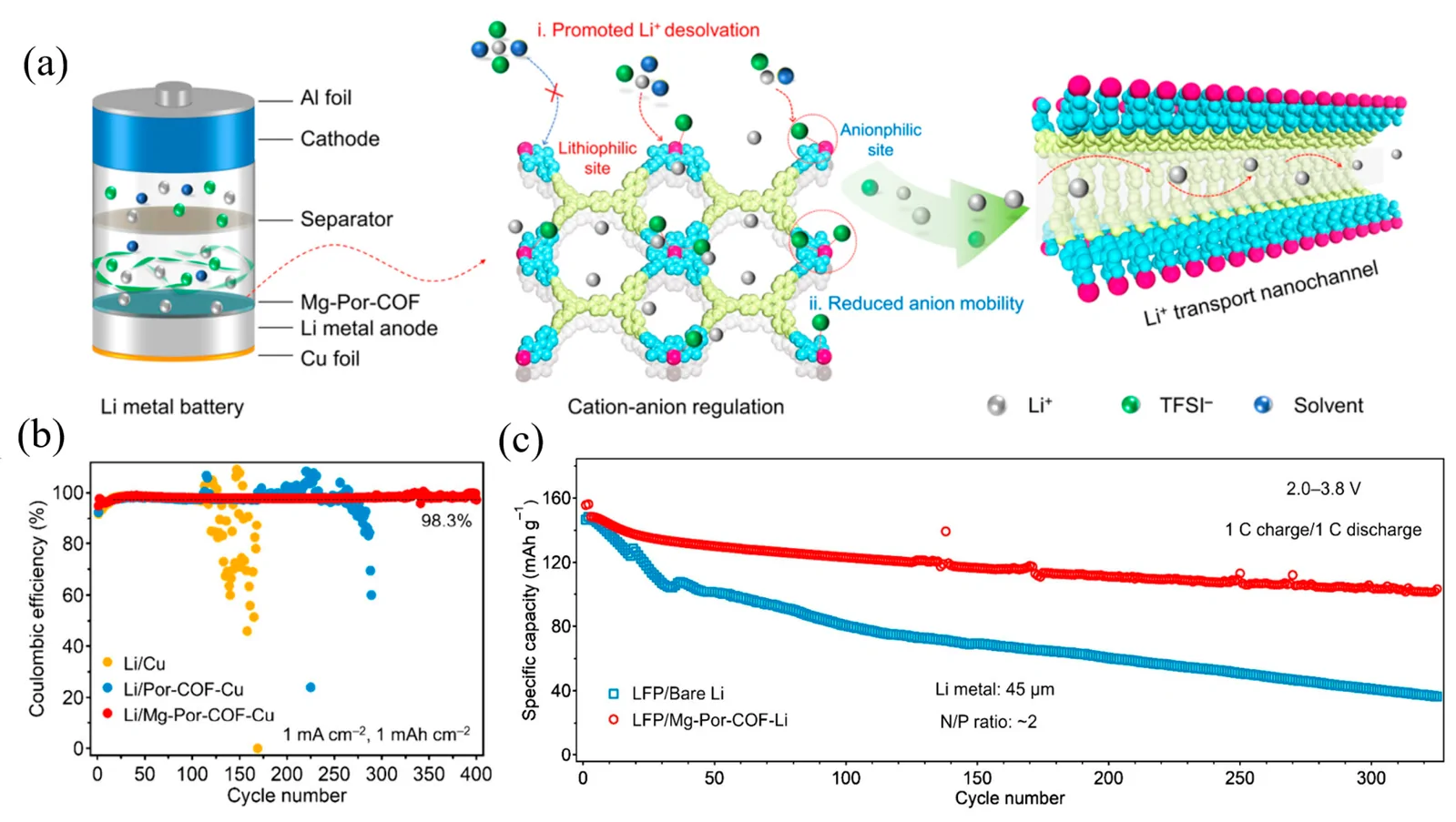
(**a**) Schematic illustration of cation–anion regulation by the COF protective layer for a Li metal battery. (**b**) Coulombic efficiencies of Li/Cu, Li/PorCOF-Cu, and Li/Mg-Por-COF-Cu cells at 1 mA cm^−2^ and 1 mAh cm^−2^. (**c**) Cycling performance of LFP full cells with a Mg-Por-COF protective layer [59]. Copyright 2025 American Chemical Society.

Zeng et al. [59] designed a magnesium porphyrin-based COF (Mg-Por-COF) as a protective layer for lithium metal anodes (Figure 4a). The core design principle lies in the synergistic regulation of both cations (Li^+^) and anions (TFSI^−^): the porphyrin rings and imine bonds in the Mg-Por-COF framework provide abundant lithiophilic sites that promote Li^+^ desolvation through strong interactions; simultaneously, the Mg^2+^ centers embedded in the porphyrin rings act as anionphilic sites, effectively anchoring TFSI^−^ anions in the electrolyte through coordination. This dual mechanism prevents space charge accumulation caused by local anion depletion, enabling smooth and dense lithium deposition even at high areal current densities of 10 mA cm^−2^. Electrochemical tests showed that the Li/Mg-Por-COF-Cu cell achieved a long cycle life of 400 cycles with an average Coulombic efficiency of 98.3% (Figure 4b), approximately 400% higher than that of bare copper foil; the LiFePO_4_/Mg-Por-COF-Li full cell maintained an average Coulombic efficiency of 99.1% over 324 cycles (Figure 4c). Theoretical calculations further confirmed the dual role of Mg-Por-COF in regulating Li^+^ transport and immobilizing TFSI^−^ anions.

This work demonstrates the significant application prospects of porphyrin-based MCOFs as protective layers for lithium metal anodes. The “lithiophilic/anionphilic dual-site design” strategy provides an important reference for interface engineering in lithium metal batteries. Currently, research on applying porphyrin/phthalocyanine-based MCOFs to lithium metal anode protection is still in its infancy. Future efforts may focus on expanding the range of metal centers (e.g., Co, Ni, Zn) and systematically investigating the influence of different metals on anionphilic capability and interfacial stability.

#### 4.1.3. Lithium-Sulfur Batteries

Lithium-sulfur batteries (LSBs) are considered promising candidates for next-generation high-energy-density storage systems due to their exceptionally high theoretical specific capacity (1672 mAh g^−1^) and energy density (2600 Wh kg^−1^) [60,61]. However, the shuttle effect of soluble lithium polysulfides (LiPSs), the insulating nature of sulfur cathodes, and volume expansion during charge–discharge processes severely constrain their cycling stability and practical application [62]. Porphyrin/phthalocyanine-based MCOFs, leveraging the strong chemical adsorption of polar M–N_4_ sites toward LiPSs and the d-orbital activation effects of metal centers on S–S bonds, can simultaneously achieve chemical anchoring of LiPSs and the promotion of conversion kinetics, making them promising candidate systems as sulfur cathode host materials.

**Figure 5:**
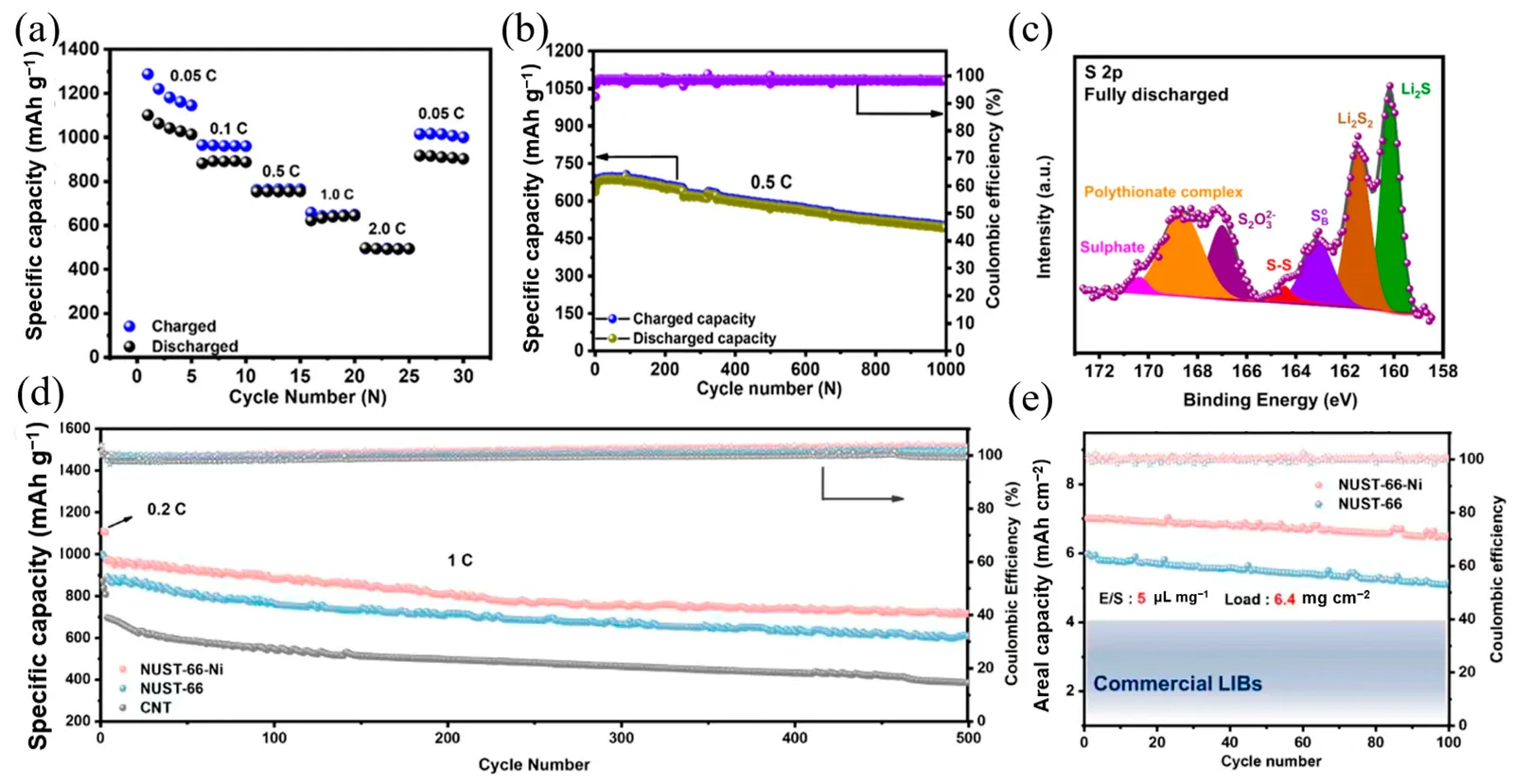
(**a**) Rate performance of S@COF-366-Co from 0.05 C to 2.0 C. (**b**) Cycling stability and Coulombic efficiency of S@COF-366-Co after 1000 cycles at 0.5 C. (**c**) High-resolution XPS of S@COF-366-Co cathode: fully discharged after 5 galvanostatic charge–discharge cycles [63]. Distributed under the terms of the Creative Commons Attribution License (CC BY 4.0). (**d**) Cycling performance of NUST-66, NUST-66-Ni, and CNT at 1 C. (**e**) Cycling performance of batteries based on NUST-66 and NUST-66-Ni under high sulfur loading at 0.2 C [64]. Copyright 2024 American Chemical Society.

El-Kaderi et al. [63] systematically compared the performance of metal-free COF-366 and Co-coordinated COF-366-Co as sulfur cathode hosts. The introduction of Co–N_4_ sites significantly enhanced high-rate electrochemical performance: S@COF-366-Co maintained a capacity of 495 mAh g^−1^ at 2 C (Figure 5a), whereas S@COF-366 failed to function above 1.5 C. After 1000 cycles at 0.5 C, S@COF-366-Co retained 493.3 mAh g^−1^ (Figure 5b), with a capacity decay rate of only 0.0345% per cycle. XPS analysis revealed that Co sites efficiently catalyze the conversion of long-chain polysulfides (Li_2_S_x_, x = 4–8) to short-chain Li_2_S_2_/Li_2_S while enhancing the chemical adsorption of polysulfides (Figure 5c). Electrochemical impedance spectroscopy demonstrated that the charge transfer resistance of COF-366-Co was substantially lower than that of the metal-free counterpart, confirming the critical role of Co–N_4_ sites in reducing interfacial impedance. Zhang et al. [64] upgraded the strategy to a “physical confinement + chemical adsorption + electrocatalysis” triple-function design, constructing 3D COFs (NUST-66 and NUST-66-Ni) based on flexible cyclooctathiophene (COTh) and porphyrin units. The 3D interconnected nanostructure provided buffering space for sulfur volume expansion, porphyrin units offered chemical adsorption sites, and Ni-porphyrin active centers accelerated Li_2_S nucleation and polysulfide conversion kinetics. This material enabled Li-S batteries with only 0.05% capacity decay per cycle over 500 cycles at 1 C (Figure 5d), far superior to commercial carbon nanotubes; under high sulfur loading (6.4 mg cm^−2^) and lean electrolyte conditions (E/S = 5 μL/mg), an areal capacity of 7.0 mAh cm^−2^ was achieved (Figure 5e), comparable to commercial lithium-ion batteries.

The application value of porphyrin/phthalocyanine-based MCOFs in lithium-sulfur batteries can be summarized in three aspects: (i) chemical anchoring-polar M–N_4_ sites and framework heteroatoms anchor LiPSs via Lewis acid–base interactions, suppressing the shuttle effect; (ii) kinetic promotion-metal centers reduce polysulfide conversion energy barriers through d-p orbital hybridization, with d-electron back-donation effectively weakening S–S bonds and accelerating the conversion of long-chain LiPSs to short-chain Li_2_S_2_/Li_2_S; and (iii) spatial confinement—ordered pore structures provide physical constraints for sulfur and its intermediates, buffering volume expansion. These three effects synergistically enhance the cycling stability and active material utilization of sulfur cathodes. Current studies have preliminarily confirmed the effectiveness of this material class as sulfur hosts. However, the influence of different metal species (Fe, Co, Ni, Cu, etc.) on catalytic pathways and selectivity remains to be systematically elucidated. Moreover, most studies remain at the coin-cell level, with pouch-cell validation and long-term cycling stability evaluation under practical conditions still very limited. Additionally, current research in the Li-S battery field is predominantly focused on porphyrin-based MCOFs systems, while the potential of phthalocyanine-based MCOFs as sulfur host materials remains to be further explored.

#### 4.1.4. Lithium-Carbon Dioxide Batteries

**Figure 6:**
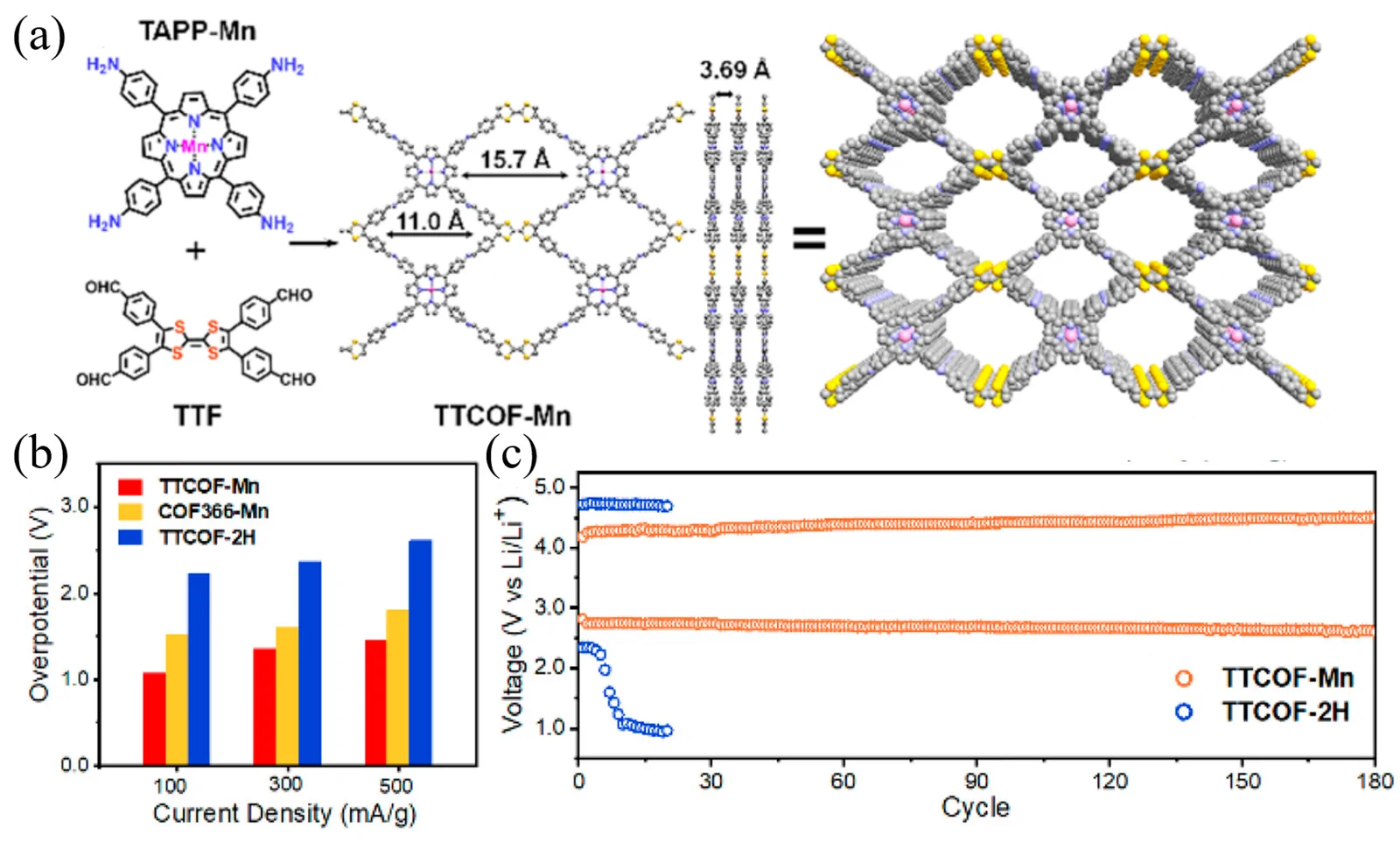
(**a**) Synthesis route and building blocks of TTCOF-Mn and its top and side viewed structure. (**b**) Overpotentials of different COF cathode catalysts under current densities of 100 mA g^−1^, 300 mA g^−1^, and 500 mA g^−1^. (**c**) Long-term cycling of the TTCOF-Mn cathode catalyst and TTCOF-2H cathode catalyst at a current density of 300 mA g^−1^ [67]. Copyright 2021 American Chemical Society.

Nonaqueous lithium-carbon dioxide (Li-CO_2_) batteries can simultaneously achieve CO_2_ fixation and utilization while delivering electrical energy, representing a novel electrochemical system with dual “carbon emission reduction” and “energy storage” functions [65,66]. However, the chemical inertness of CO_2_ molecules and the difficulty of decomposing the discharge product Li_2_CO_3_ result in extremely high overpotentials and poor cycling stability. Efficient cathode catalysts are key to achieving CO_2_ reduction and evolution reactions. Lan et al. [67] synthesized a porphyrin-based COF (TTCOF-Mn) via Schiff-base condensation using the electron donor tetrathiafulvalene (TTF) and tetrakis(4-aminophenyl)porphyrin manganese (TAPP-Mn) as building units. This material features dual micropore channels (pore sizes of 1.10 and 1.57 nm) (Figure 6a) and uniformly dispersed single Mn(II) active sites. Electrochemical tests revealed that the TTCOF-Mn cathode catalyst exhibited an overpotential as low as 1.07 V at 100 mA g^−1^ (Figure 6b) and maintained stable cycling for 180 cycles at 300 mA g^−1^ (Figure 6c). Notably, comparative experiments showed that only when the porphyrin center was Mn did the battery display a discharge plateau around 2.9 V, whereas other metal porphyrin sites (Co, Ni, Cu) exhibited discharge plateaus around 2.5 V. DFT calculations revealed that Mn sites could achieve efficient reduction and Li_2_CO_3_ decomposition via a four-electron CO_2_ conversion pathway (4Li^+^ + 3CO_2_ + 4e^−^ → 2Li_2_CO_3_ + C), while other metal sites followed a two-electron pathway. This work demonstrates the potential of porphyrin-based MCOFs as Li-CO_2_ battery cathode catalysts, with the core contribution being the revelation of the unique role of Mn–N_4_ sites in regulating CO_2_ reduction reaction pathways, while ordered pores facilitate mass transport and active site accessibility. Currently, this direction has only one systematic report, and the regulatory rules of different metal centers on reaction pathway selectivity require further exploration.

### 4.2. Zinc-Based Batteries

#### 4.2.1. Zinc-Air Batteries

Zinc-air batteries, utilizing metallic zinc as the anode and atmospheric oxygen as the cathode active material, offer advantages including high theoretical energy density, abundant resources, and environmental friendliness. However, both the oxygen reduction reaction (ORR) and oxygen evolution reaction (OER) at the air cathode involve multi-step electron transfer processes with sluggish kinetics and high overpotentials, representing performance bottlenecks [68]. The development of efficient and stable bifunctional ORR/OER catalysts is critical for the practical application of zinc-air batteries.

In phthalocyanine-based MCOFs, Liu et al. [69] synthesized a ladder-type π-conjugated two-dimensional benzimidazophenanthroline-linked iron phthalocyanine MCOF (FePc-BBLCOF) via the solvothermal condensation of 2,3,9,10,16,17,23,24-octacarboxyphthalocyanine iron (Fe-OCAP) with 1,2,4,5-tetraaminobenzoquinone (TABQ). The innovation lies in the robust fused aromatic skeleton and BBL linkages imparting excellent conductivity, along with uniformly dispersed high-activity Fe-N_4_ sites. In alkaline media, FePc-BBLCOF exhibited a half-wave potential of 0.933 V (vs. RHE), a Tafel slope of 24.8 mV dec^−1^, a turnover frequency (TOF) of 78.1 e^−^·site^−1^·s^−1^ (at 0.85 V vs. RHE), and a mass activity of 26.5 A mg^−1^ Fe, which are 16.4 and 57.2 times those of commercial Pt/C, respectively. As the air cathode catalyst in zinc-air batteries, FePc-BBLCOF achieved a maximum power density of 181.4 mW cm^−2^ and a specific capacity of 773 mAh g^−1^, both outperforming the Pt/C benchmark.

In porphyrin-based MCOFs, Zhang et al. [70] constructed a CNT@POF cathode by compositing a cobalt porphyrin MCOF (POF) with carbon nanotubes. In liquid zinc-air batteries, this cathode exhibited a charge–discharge voltage gap of only 0.71 V (lower than the 0.77 V of noble-metal-based Pt/C+Ir/C) and a peak power density of 237 mW cm^−2^ (far exceeding the 78 mW cm^−2^ of Pt/C+Ir/C). In flexible all-solid-state zinc-air batteries, it achieved an open-circuit voltage of 1.39 V and an energy efficiency of 61.6%. Yao et al. [71] designed a hydrophilic sp^2^-carbon-conjugated cobalt porphyrin MCOF (CoT-sp^2^C-P-COF-COOH) by converting cyano groups to carboxyl groups. The hydrophilic modification (contact angle reduced from 121° to 61°) promoted continuous proton-coupled electron transfer processes using water as the proton donor, while the sp^2^-carbon-conjugated backbone enhanced intrinsic conductivity. This catalyst achieved a half-wave potential of 0.823 V (vs. RHE) for ORR, and the assembled zinc-air battery exhibited a peak power density of 121.8 mW cm^−2^. Theoretical calculations indicated that carboxyl modification optimized the adsorption mode of O_2_ molecules, promoting the formation of Co^III^-O_2_^−^ intermediates and thereby accelerating PCET steps and ORR kinetics.

The above work demonstrates the application potential of phthalocyanine/porphyrin-based MCOFs as air cathode catalysts for zinc-air batteries. Their core advantages lie in the modulation of ORR oxygen-containing intermediate adsorption energies by M–N_4_ sites and the charge transport channels provided by conjugated backbones (and carbon nanotube composites). Currently, most studies focus on ORR catalytic performance, while systematic evaluation of OER performance and bifunctional integrated design remain in their infancy.

#### 4.2.2. Zinc-Iodine Batteries

**Figure 7:**
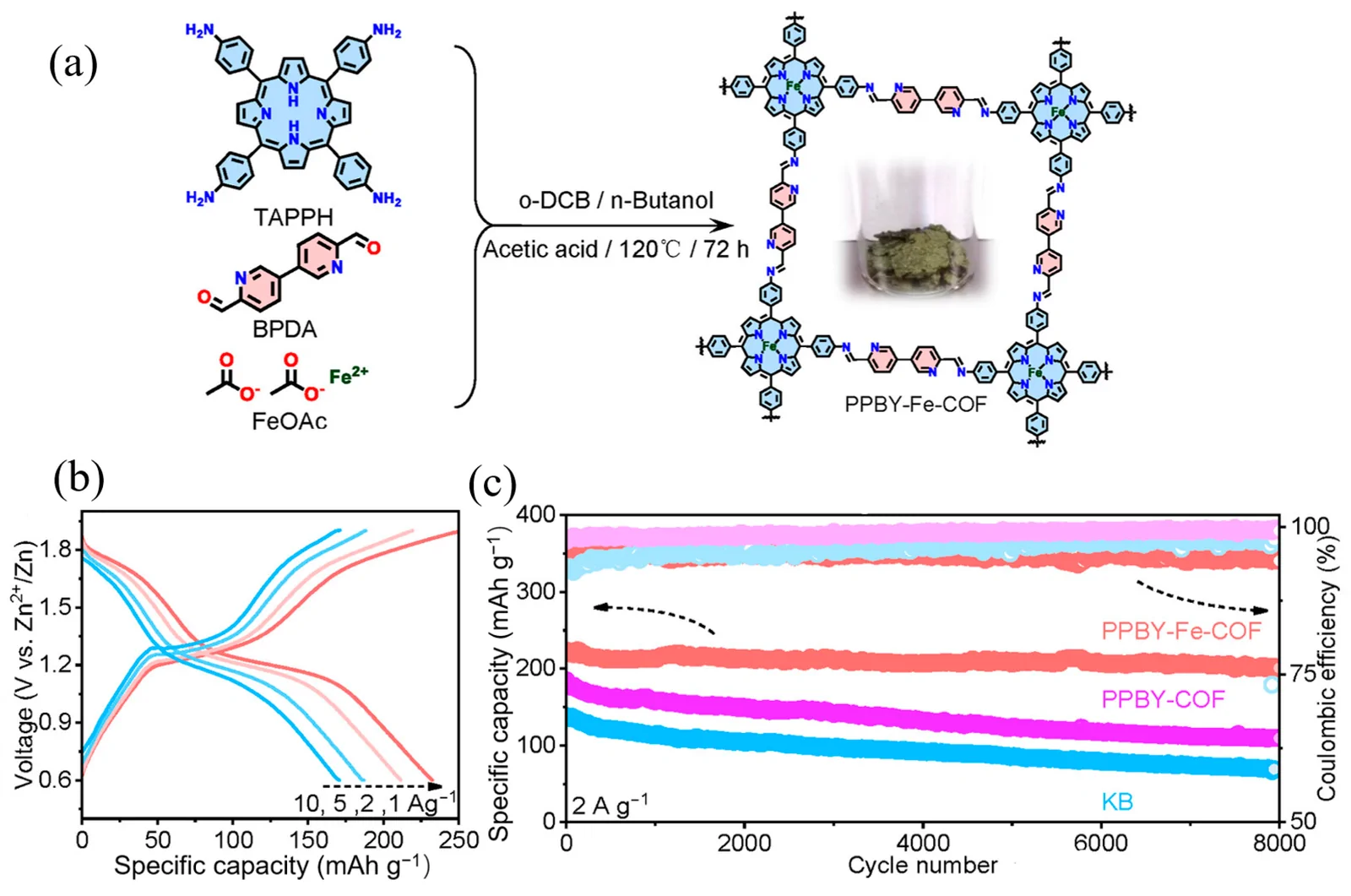
(**a**) Schematic illustration of the synthesis of PPBY-Fe-COF. (**b**) Charge–discharge curves of PPBY-Fe-COF. (**c**) Cycling performance of PPBY-Fe-COF and PPBY-COF at a current density of 2 A g^−1^ [74]. Copyright 2025 American Chemical Society.

Aqueous zinc-iodine batteries offer advantages including high theoretical specific capacity (211 mAh g^−1^), good safety, and low cost, making them promising aqueous energy storage systems [72,73]. However, poor iodine species conductivity and the polyiodide shuttle effect severely constrain cycling stability. Extending the conventional I^−^/I^0^ two-electron reaction to a I^−^ → I^0^ → I^+^ four-electron continuous oxidation could theoretically double the specific capacity (from 211 mAh g^−1^ to 422 mAh g^−1^), but the hydrolysis tendency of the highly reactive I^+^ intermediate places higher demands on host materials for anchoring and stabilization. Han et al. [74] designed an iron-coordinated porphyrin bipyridine MCOF (PPBY-Fe-COF) as an iodine host material (Figure 7a), achieving four-electron transfer through the synergistic division of labor between Fe–N_4_ sites (cationic sites) and C=N groups (anionic sites): Fe sites facilitate I^−^/I^0^ oxidation, while C=N sites capture I^0^ and further oxidize it to I^+^, with OTF^−^ assisting the formation of N-I^+^-O bonds to effectively suppress I^+^ hydrolysis. This battery delivered a specific capacity of 240 mAh g^−1^ (based on iodine mass) at 1 A g^−1^ (Figure 7b), with 90.9% capacity retention after 8000 cycles (Figure 7c) and an energy density of 294.4 Wh kg^−1^, approximately twice that of conventional two-electron systems. This work demonstrates the potential of porphyrin-based MCOFs as iodine host materials for zinc-iodine batteries, with the core contribution being the provision of a feasible catalytic and stabilization strategy for overcoming the capacity limitations of traditional two-electron reactions through the functional division of Fe–N_4_ and C=N dual sites. Research in this direction is still in its infancy.

### 4.3. Sodium/Potassium-Ion Batteries

Sodium and potassium are considered promising complements to lithium-ion batteries due to their abundant resources and low cost, particularly suitable for large-scale energy storage applications. However, the ionic radii of Na^+^ (1.02 Å) and K^+^ (1.38 Å) are substantially larger than that of Li^+^ (0.76 Å), making it difficult for conventional graphite anodes to achieve efficient reversible storage of these large ions, leading to sluggish electrode kinetics and rapid capacity decay. In inorganic anode systems, heterostructure engineering is often employed to address these kinetic and stability issues. [75] For COFs, the designable pores of COFs provide a structural basis for large-ion storage, though the conductivity and active site density of conventional COFs remain to be improved. Introducing metal ions into porphyrin/phthalocyanine macrocycles to form MCOFs can utilize the redox activity of M–N_4_ sites to contribute additional capacity, while the charge transport capability of conjugated backbones can improve rate performance.

**Figure 8:**
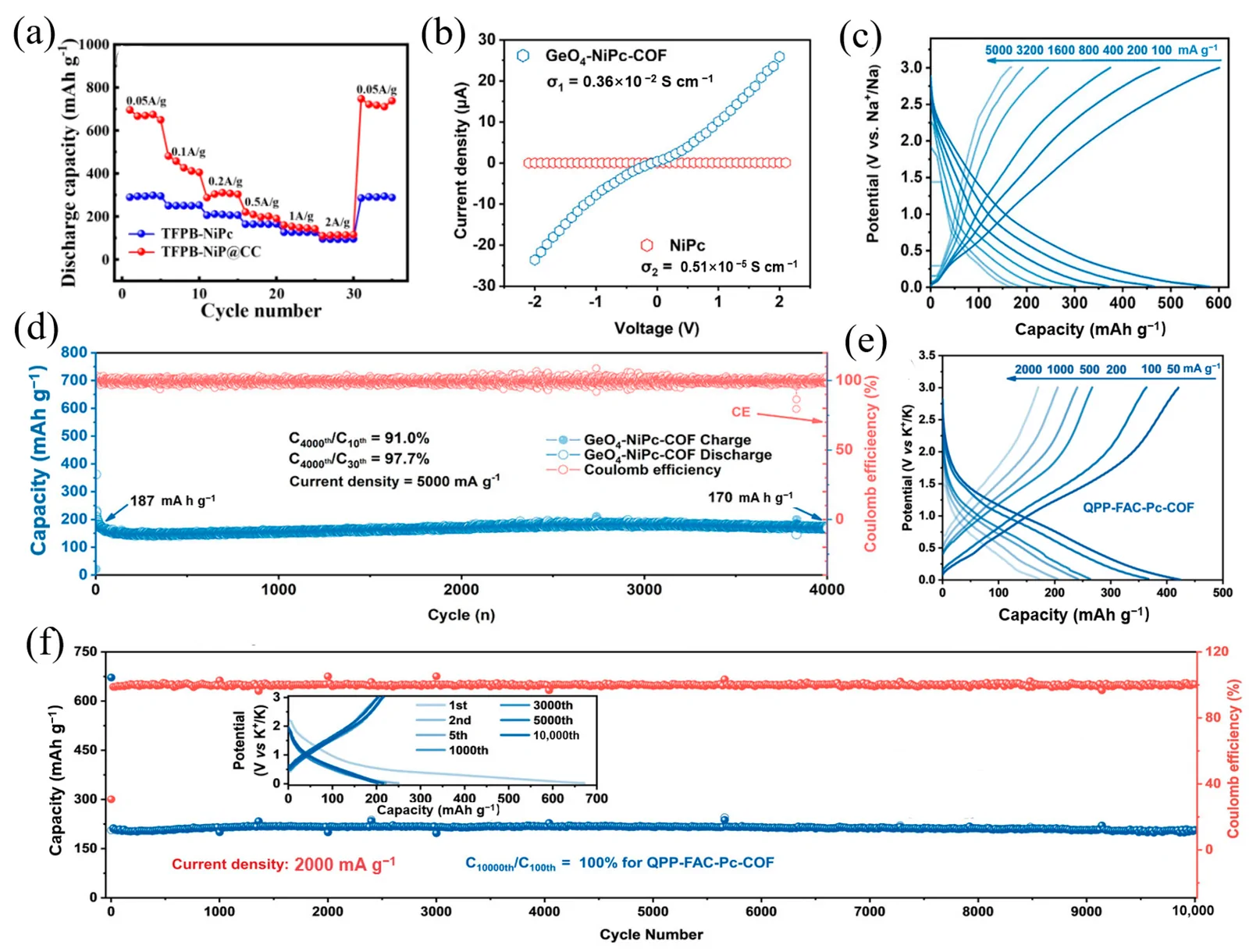
(**a**) Rate performance of TFPB-NiPc and TFPB-NiPc@CC electrodes [53]. Copyright 2025 American Chemical Society. (**b**) Current-voltage curves of GeO_4_-NiPc-COF and NiPc. (**c**) Charge–discharge curves of GeO_4_-NiPc-COF. (**d**) Cycling stability of GeO_4_-NiPc-COF at 5000 mA g^−1^ [76]. Copyright 2025 Wiley-VCH GmbH. (**e**) Charge–discharge curves of QPP-FAC-Pc-COF at various current densities. (**f**) Long-term cycling performance of QPP-FAC-Pc-COF at 2000 mA g^−1^ [77]. Copyright 2022 Wiley-VCH GmbH.

For sodium-ion batteries, Chen et al. [53] grew phthalocyanine MCOFs (TFPB-NiPc) on carbon cloth in situ, obtaining a binder and additive-free self-supporting composite anode TFPB-NiPc@CC. At a current density of 0.05 A g^−1^, TFPB-NiPc@CC delivered a discharge specific capacity of 695.5 mAh g^−1^ (Figure 8a), substantially higher than the 298.4 mAh g^−1^ of pristine TFPB-NiPc, demonstrating that the introduction of carbon cloth effectively enhances electrochemical performance. Liu et al. [76] further prepared 2D phthalocyanine COFs (GeO_4_-MPc-COFs) by reacting octahydroxyphthalocyanine metal complexes MPc(OH)_8_ (M = Co, Ni, Zn) with GeO_2_. The p–π interactions between the planar Ge(IV)O_4_ linkers and phthalocyanine macrocycles promoted π–electron delocalization throughout the 2D network, yielding conductivities of 0.14–0.36 × 10^−2^ S cm^−1^ (Figure 8b). The synergistic effect of the reversible redox activity of Ge(IV)O_4_ linkers and the multiple active sites of phthalocyanine units enabled GeO_4_-NiPc-COF to achieve a reversible specific capacity of 607 mAh g^−1^ at 100 mA g^−1^ (Figure 8c), with only 0.00057% capacity decay per cycle after 4000 cycles at 5 A g^−1^ (Figure 8d).

For potassium-ion batteries, Jiang et al. [77] synthesized two fully aromatic conjugated phthalocyanine COFs (BB-FAC-Pc-COF and QPP-FAC-Pc-COF) via a solvent-free ionothermal method, both featuring sql topology and AA stacking, with conductivities of 0.93–1.94 × 10^−4^ S cm^−1^. QPP-FAC-Pc-COF delivered a reversible capacity of 424 mAh g^−1^ at 50 mA g^−1^ (Figure 8e), maintaining nearly 100% capacity retention after over 10,000 cycles at 2000 mA g^−1^ (Figure 8f). Subsequently, the same group [78] employed octacarboxy-substituted tetrapyrazinoporphyrazine cobalt(II) [CoTPyzPA(COOH)_8_] as the organic building unit, reacting with p-phenylenediamine (PD) and 4,4′-biphenyldiamine (BD) via hydrothermal synthesis to produce two crystalline polyimide COFs—TPyzPA-PD-COF and TPyzPA-BD-COF. The three types of nitrogen atoms in the tetrapyrazinoporphyrazine macrocycle, together with the carbonyl oxygen atoms of imide bonds, constitute abundant accessible redox-active sites. As potassium-ion battery anodes, TPyzPA-PD-COF exhibited a reversible capacity of 467 mAh g^−1^ at 50 mA g^−1^ and maintained nearly 100% capacity retention after 3500 cycles at 2 A g^−1^; in contrast, TPyzPA-BD-COF delivered a capacity of 385 mAh g^−1^ under the same conditions due to the presence of more inert biphenyl units. This comparison demonstrates that linker length and conjugation degree significantly influence potassium storage capacity—shorter p-phenylenediamine linkers favor higher active site density and better charge transport efficiency.

In summary, the application value of porphyrin/phthalocyanine-based MCOFs in sodium/potassium-ion batteries can be understood at two levels: (i) promotion of ion transport and active site accessibility by ordered pores—the well-defined pores of the COF framework facilitate the diffusion of large ions while exposing active sites on pore surfaces for improved utilization; and (ii) synergistic energy storage from multiple redox-active sites—the phthalocyanine/porphyrin macrocycles and M–N_4_ metal centers provide abundant redox sites, with some linkers also exhibiting reversible redox activity, all three contributing synergistically to capacity.

In terms of industrialization prospects, lithium-sulfur and zinc-air batteries exhibit the highest application potential for porphyrin/phthalocyanine MCOFs, benefiting from low raw material costs and the exclusive capacity of M–N_4_ sites to tackle core obstacles including polysulfide shuttle effect and sluggish oxygen redox kinetics. Sodium and potassium ion batteries are suitable for large-scale stationary energy storage owing to abundant resource reserves, whereas Li-CO_2_ and zinc-iodine batteries are confined to niche applications and fundamental research at the current stage.

### 4.4. Supercapacitors

Supercapacitors have attracted widespread attention due to their fast charge–discharge capability, long cycle life, and high power density, with performance highly dependent on the electrochemical characteristics of electrode materials [79,80]. Two-dimensional layered structures facilitate rapid ion transport between layers, while three-dimensional porous frameworks provide richer ion transport channels and active surfaces. Meanwhile, macrocyclic conjugated backbones contribute to in-plane charge transport, and the reversible redox reactions of metal centers can contribute pseudocapacitance. These characteristics endow porphyrin/phthalocyanine-based MCOFs with unique advantages in the supercapacitor field.

In porphyrin-based MCOFs, Jiang et al. [81] prepared a porphyrin COF self-supporting nanofilm (COF-TAPP-DHPA) via surfactant monolayer-assisted interfacial synthesis. After Co^2+^ chelation and ionic liquid grafting co-modification, the film was directly patterned into interdigitated electrodes and assembled into planar micro-supercapacitors. This device achieved an energy density of 67.2 mWh cm^−3^ at a power density of 3.46 W cm^−3^, with 91% capacitance retention after 5000 cycles.

In phthalocyanine-based MCOFs, Sun et al. [82] prepared a CoPc-COF/chitosan self-supporting aerogel electrode via freeze-drying. The porous network provided abundant active surfaces and short ion diffusion paths, with surface nitrogen-containing groups and Co–N_4_ centers jointly enhancing pseudocapacitive contribution. This aerogel delivered a specific capacitance of 556 F g^−1^ at 1 A g^−1^; the assembled asymmetric supercapacitor achieved an energy density of 31.9 Wh kg^−1^ at a power density of 750 W kg^−1^, with 87.5% capacitance retention after 5000 cycles. Chen et al. [83] synthesized ultrathin CoPPc nanosheets with a thickness of approximately 1.6 nm via a mild solid-state method. Their excellent electrochemical storage capability (165 F g^−1^ at 0.25 A g^−1^) originates from the synergistic contribution of electric double-layer capacitance from the ultrathin nanosheets and pseudocapacitance from reversible cobalt-center redox reactions. The CoPPc-based quasi-solid-state flexible symmetric supercapacitor achieved an energy density of 39.8 Wh kg^−1^, with 104.3% capacitance retention after 30,000 cycles and 80.97% retention after 10,000 bending tests.

In summary, the application mechanisms of porphyrin/phthalocyanine-based MCOFs in supercapacitors can be categorized into two aspects: (i) electric double-layer capacitance contribution from ordered layered/porous structures-2D layered structures facilitate rapid electrolyte ion diffusion, while 3D porous frameworks provide richer ion transport channels and active surfaces; and (ii) pseudocapacitive contribution from metal centers and heteroatom sites-reversible redox reactions of M–N_4_ sites and framework heteroatoms (N, O, etc.) can contribute additional capacity. Current studies have preliminarily validated the feasibility of this material class in supercapacitors, particularly demonstrating unique advantages in flexible and miniaturized devices.

The electrochemical parameters of representative MCOFs electrode materials for diverse energy storage systems are compiled in Table 2.

## 5. Conclusions and Outlook

This review systematically surveyed recent research progress on porphyrin/phthalocyanine-based MCOFs in terms of synthetic strategies, dimensional regulation, and electrochemical energy storage applications. As an emerging class of porous crystalline materials, porphyrin/phthalocyanine-based MCOFs effectively integrate the metal active sites of MOFs with the high chemical stability imparted by strong covalent bonds in COFs, while retaining the high specific surface area and tunable porosity of both material classes. Through the synergistic effects of macrocyclic conjugated backbones and M–N_4_ sites, they demonstrate unique potential in electrochemical energy storage.

Regarding synthetic strategies, three pathways—pre-metallation, simultaneous metallation, and post-metallation—have been well-established. Pre-metallation enables highly uniform dispersion of metal sites and consistent coordination geometries, but involves complex, time-consuming procedures. Simultaneous metallation combines metal coordination and framework construction into a single step, significantly improving synthetic efficiency and scalability, though reaction conditions must be finely tuned to avoid uneven coordination. Post-metallation offers the greatest flexibility, facilitating multi-metal functionalization screening of the same precursor, but metal loading and distribution uniformity are limited by diffusion processes. Each strategy has its own applicable scenarios, and researchers should choose flexibly based on target material systems and performance requirements. In terms of dimensional regulation, 2D structures provide efficient charge transport channels through in-plane conjugation and interlayer π–π stacking, suitable for scenarios requiring high-rate performance; 3D structures enhance framework stability through spatial interlocking and can offer multidirectional ion transport pathways under appropriate design, holding application potential in energy storage systems requiring long cycle life. In energy storage applications, this class of materials has demonstrated multifaceted functional advantages in lithium-based batteries (lithium-ion, lithium-sulfur, lithium-CO_2_, lithium metal protection), zinc-based batteries (zinc-air, zinc-iodine), sodium/potassium-ion batteries, and supercapacitors. Their mechanisms of action can be summarized as catalytic conversion, chemical anchoring/confinement, interface stabilization, and pseudocapacitive contribution.

Despite the progress outlined above, research on porphyrin/phthalocyanine-based MCOFs remains in a stage of rapid development but is far from mature. The challenges that lie ahead can be broadly categorized into two types: those inherited from the general COF/MOF platform, such as poor intrinsic conductivity, limited scalability of solvothermal synthesis, and insufficient in situ mechanistic characterization (points 1, 3, and 5 below)—and those uniquely associated with the porphyrin/phthalocyanine macrocyclic system (points 2 and 4 below). These intrinsic material challenges are further compounded by practical considerations regarding performance evaluation and sustainability (points 6 and 7). Future breakthroughs should focus on the following directions:

(1) Enhancement of intrinsic conductivity. Most MCOFs currently fall within the semiconductor or insulator category, severely limiting high-rate performance. The development of fully sp^2^-carbon conjugated frameworks, the introduction of electron-deficient units such as triazine to construct donor–acceptor-type structures, and the exploration of interlayer charge transfer synergistic mechanisms represent viable pathways for improving intrinsic conductivity.
(2) Optimization of active site utilization. For porphyrin/phthalocyanine macrocycles, two molecular-level factors are particularly relevant: the peripheral substituents dictate solubility and assembly behavior, while the planarity of the macrocyclic core governs interlayer stacking order, both of which critically influence the accessibility of active sites. Chemical functionalization of pore microenvironments (e.g., introducing polar groups or tuning nucleophilicity/electrophilicity) can optimize ion desolvation behavior and intermediate adsorption energies, thereby enhancing site accessibility and reaction selectivity. Additionally, reducing interlayer stacking density or introducing mesoporous/macroporous hierarchical structures can further expose active sites buried between layers.
(3) Green and scalable synthesis for macrocyclic systems. Current solvothermal methods cannot meet the industrial demands in terms of yield, energy consumption, and reproducibility. Developing aqueous-phase synthesis, solvent-free mechanochemical methods, or continuous flow processes, with adaptation optimization for the solubility and reactivity of porphyrin/phthalocyanine macrocycles, is key to achieving scalable production.
(4) Precise regulation of metal center electronic states and structure–property relationship elucidation. Different metals (Fe, Co, Ni, Cu, Mn, etc.) exhibit significant differences in d-electron configurations and d-band center positions, which substantially affect catalytic pathways and reaction selectivity. Through ligand-field tuning, peripheral substituent modification, or bimetallic synergistic strategies, targeted optimization of metal site catalytic activity can be achieved. Concurrently, combining in situ characterization techniques (such as in situ XAS and in situ Raman) to deeply reveal the dynamic evolution of metal valence states, coordination numbers, and local structures during charge–discharge processes will provide direct evidence for validating and iteratively optimizing the aforementioned design strategies.
(5) Device-level performance validation and failure mechanism studies. Currently, most studies remain at the coin-cell level, with pouch-cell, high-loading electrode, and practical-condition long-term cycling evaluations extremely limited. There is an urgent need to establish a “material-electrode-device” multi-scale evaluation system to systematically investigate structural degradation pathways and the capacity fading origins of MCOFs under complex operating conditions.
(6) Standardized performance evaluation protocols. The wide disparity in testing conditions (e.g., current densities, electrolyte compositions, and electrode loadings) across different reports complicates direct cross-material comparison. Establishing unified benchmarking standards is essential for objectively assessing the true potential of MCOFs relative to other electrode systems.
(7) Sustainability considerations for metal selection. While MCOFs offer a pathway toward less resource-intensive energy storage, the cost, toxicity, and environmental impact of metal precursors (particularly Co and Ni) have not been systematically addressed. Future efforts should explore earth-abundant and low-toxicity alternatives (e.g., Fe, Mn, Zn) to align materials development with practical sustainability requirements.

In conclusion, porphyrin/phthalocyanine-based MCOFs, as structurally designable and functionally tailorable advanced materials, hold broad prospects in electrochemical energy storage. Future research requires coordinated advancement at the levels of molecular design, controllable synthesis, mechanistic understanding, and device integration. Interdisciplinary methodological integration—such as AI-assisted synthesis condition optimization and structural screening, in situ characterization revealing dynamic structure-property relationships, and engineering exploration toward practical requirements—is expected to accelerate the translation of this material class from laboratory to practical application.

## Figures and Tables

**Table 1:** Comparison of three metallation routes for MCOFs.

Metallation Route	Metal Site Uniformity	Synthetic Cost & Cycle	Metal Loading Tunability	Typical Optimal Application Scenarios
Pre-metallation	Excellent	High cost, long synthesis time	High, fixed single metal	Devices requiring highly consistent M–N_4_ catalytic sites
Simultaneous metallation	Medium (risk of competitive coordination)	Low cost, one-pot rapid synthesis	Medium, co-doped multi-metal	Large-scale mass production
Post-metallation	Medium (surface metal aggregation risk)	Medium, flexible modular synthesis	Wide range, sequential multi-metal doping	Systematic metal screening research, bimetallic MCOFs

**Table 2:** Electrochemical performance summary of typical MCOFs electrodes for various energy storage systems.

Material	Metallation Strategy	Energy Storage System	Test Current Density	Specific Capacity	Long-Cycle Performance	Ref.
CoPc-3Ph-COF	Pre-metallation	Lithium-ion anode	100 mA g^−1^	1086 mAh g^−1^	/	[52]
TFPB-NiPc@CC	Pre-metallation	Lithium-ion anode	200 mA g^−1^	1090.2 mAh g^−1^	994.5 mAh g^−1^ after 500 cycles	[53]
PMDA-NiPc	Pre-metallation	Lithium-ion anode	100 mA g^−1^	1861 mAh g^−1^	Full cell retains 74.7% capacity after 200 cycles@1C	[54]
Mg-Por-COF	Post-metallation	Lithium metal anode	1 mA cm^−2^	/	Stable 400 cycles	[59]
S@COF-366-Co	Pre-metallation	Lithium-sulfur cathode	0.5 C	/	Capacity decay rate: 0.0345% per cycle	[63]
NUST-66-Ni	Pre-metallation	Lithium-sulfur cathode	1 C	/	0.05% decay per cycle over 500 cycles	[64]
TTCOF-Mn	Pre-metallation	Li-CO_2_ battery cathode catalyst	300 mA g^−1^	/	Stable cycling for 180 cycles	[67]
FePc-BBLCOF	Pre-metallation	Zinc-air battery bifunctional catalyst	/	Peak power density: 181.4 mW cm^−1^	Superior ORR/OER bifunctional activity vs. Pt/C	[69]
CNT@POF	Pre-metallation	Zn-air cathode	/	Peak power density: 237 mW cm^−2^	Solid-state device energy efficiency 61.6%	[70]
PPBY-Fe-COF	Post-metallation	Aqueous zinc-iodine cathode	1 A g^−1^	240 mAh g^−1^	90.9% capacity retention after 8000 cycles	[74]
GeO_4_-NiPc-COF	Pre-metallation	Sodium-ion anode	100 mA g^−1^	607 mAh g^−1^	0.00057% decay per cycle over 4000 cycles at 5 A g^−1^	[76]
QPP-FAC-Pc-COF	Simultaneous metallation	Potassium-ion anode	50 mA g^−1^	424 mAh g^−1^	Near 100% capacity retention over 10,000 cycles at 2 A g^−1^	[77]
CoPc/chitosan aerogel	Pre-metallation	Symmetric supercapacitor	1 A g^−1^	556 F g^−1^	87.5% capacitance retention after 5000 cycles	[82]
Ultrathin CoPPc nanosheet	Pre-metallation	Flexible quasi-solid supercapacitor	0.25 A g^−1^	165 F g^−1^	104.3% retention after 30,000 cycles	[83]

## Data Availability

No new data were created or analyzed in this study. Data sharing is not applicable to this article.
